# Comparative Study of the Dimensional and Shape Accuracy of Parts Made of 316L Manufactured Using the SLM and Casting Methods after Milling and WEDM

**DOI:** 10.3390/ma17122907

**Published:** 2024-06-14

**Authors:** Magdalena Machno, Wojciech Zębala

**Affiliations:** 1 Department of Rail Vehicles and Transport, Faculty of Mechanical Engineering, Cracow University of Technology, 31-155 Cracow, Poland; 2 Department of Production Engineering, Faculty of Mechanical, Cracow University of Technology, 31-155 Cracow, Poland; wojciech.zebala@pk.edu.pl

**Keywords:** additive manufacturing, selective laser melting, stainless 316L, milling, wire electrical discharge machining

## Abstract

Parts made using selective laser melting (SLM) often require improvements to the quality of side surfaces. Therefore, the analysis of the machinability of metallic printed material is new/innovative. The surface of printed parts requires improvement in quality—surface roughness. Hence, there is a need for effective manufacturing techniques that improve the quality of the side surfaces of printed parts. In our work, we try to fill this research gap. This work comparatively analyzed the surface quality (roughness parameter Ra) after milling and wire electrical discharge machining (WEDM). The processed material was AISI 316L stainless steel, which was produced using the casting and SLM method. In the case of printed material, the influence of the direction of the tool (perpendicular, parallel) on the arrangement of sintered layers was also analyzed. The analysis of the results showed that processing the cast material and processing the material perpendicular to the arrangement of the layers gives similar results—similar relationships between the processing parameters and surface roughness were observed. However, processing parallel to the arrangement of sintered layers showed ambiguity in the relationships. Moreover, the best results of the Ra parameter (0.1–0.2 µm) were obtained for feeds of 0.08 mm/rev and 0.12 mm/rev and a cutting speed of 90 m/min. In this work, the novelty is the comparison of the surfaces of materials manufactured using different techniques (SLM, casting) after milling and WEDM processing.

## 1. Introduction

Stainless steel alloys are widely used in many industries such as aerospace, medical device, pipeline, automotive, and die and tool industries. One of the most popular stainless steels is AISI 316L steel, which is an austenitic alloy widely used in industrial applications due to its high corrosion resistance with acceptable mechanical properties [1,2]. Additionally, austenitic steels are classified as difficult-to-cut, which is why additive manufacturing (AM) techniques have recently become an alternative to producing parts from them [3]. AM technology has become increasingly popular in parts manufacturing technology in recent years. Additive methods are used more and more often in various industries [4,5]. They make it possible to make parts with complex geometry from modern engineering materials (such as titanium alloys, nickel–chromium superalloys, super-durable steels). One of the additive methods is selective laser melting (SLM), i.e., the laser sintering of metallic powders layer by layer [6,7]. SLM technology makes it possible to print parts with good dimensional and shape accuracy, i.e., made at the shaping stage. However, the quality of the side surfaces of printed parts often requires finishing [8,9]. When selecting finishing processing, the properties of the material from which the part is printed and the geometry of the part should be taken into account. After laser sintering, the properties of the metal powder change, and the structure of the sintered material is different from the cast material. One approach to better study the machining of cast material can be achieved by comparison with the machining of cast materials [10]. Additionally, the advantage of the material printed using the SLM method is reducing the weight of the part by approximately 60% [8].

The SLM manufacturing process uses a laser beam to selectively melt metal powder layer by layer. Each layer of molten metal is deposited on top of the previous layer [11]. Because the molten material in each layer is rapidly melted and then cooled by the surrounding powder, the geometry of the printed parts may need to be improved. The sintered material is characterized by microstructural features such as anisometric grains, strong textures, significant residual dislocations, solidification substructure, and chemical segregation, which are therefore typical of finished SLM parts. When comparing these non-equilibrium microstructures with cast materials, they may improve some desirable material properties, such as hardness and strength, but may impair others, such as ductility and corrosion resistance [1,12]. Moreover, the structure of the metal printed material is porous, which affects the subsequent surface quality and the selection of processing parameters to improve quality [13].

Various machining techniques can be used for the additional processing of metallic material sintered using the SLM method—conventional [14,15] and unconventional [16,17]. In [10], milling was chosen as the surface treatment of material sintered using the SLM method. The study examined the influence of milling parameters and the direction of layer growth on machining. The analysis of the results showed significant differences in the milling results, which depend on the geometry and dimensions of the paths created during the SLM process. The results of these studies show that SLM technology affects the milling process.

When milling surfaces after metallic 3D (three-dimensional) printing, material cracks may remain on the surface after processing. In [18], the test results showed the presence of cracks and breaches on the milled surface. It was shown that the depth and size of the cracks depend on the cutting edge feed and the milling method. The maximum crack depth exceeded 150 µm. Additionally, the analysis of the results showed that climb milling has a positive effect on the quality of the machined surface. However, clear traces of tool passage were observed on the machined surface. The research results from these tests show that selecting the optimal values of the parameters of the milling process of the sintered material is a challenge and requires further refinement.

Unconventional methods are also used to improve the surface quality after the selective laser sintering process. In work [16], wire electrical discharge polishing (WEDP) was used to improve the surface quality of parts after SLM printing. The analysis of the machined surface showed that the WEDP process effectively removes circularity defects and the presence of unmelted or partially melted material. It was shown that after this unconventional process, the printed surface had reduced roughness parameters Sa, Sq, and Sz. Therefore, the electroerosion process is promising for improving the surface quality of SLM-printed material. Different factors influence the machinability of sintered materials. The analysis of the machinability of 316L steel manufactured using the SLM method was carried out in [19]. The study examined the influence of finishing milling on the mechanical properties of this steel and the quality of the machined surface. The influence of machining parameters on the phase, cutting force, and surface roughness was analyzed. The influence of scanning speed on steel machinability was also analyzed. The analysis of the results showed that scanning speed affects the roughness of the machined surface and causes higher cutting forces and vibrations during machining. Various methods of processing difficult-to-cut materials were also analyzed in [20]. The machinability of titanium matrix composites was analyzed using milling and discharge electrical machining. The test results showed that the method of producing the material affects its machinability. For EDM machining, the sizes and distribution of erosion pores were different for the four types of TMC. However, in the case of milling, the analysis of the results showed that higher resultant cutting forces and stronger vibrations were found when machining SB800 and SB900 due to the significant improvement in mechanical properties.

The above analysis of these works shows that the surface treatment after printing using selective laser melting is demanding. Moreover, due to the layered structure, the presence of pores, and the influence of the orientation of the sintered layers in the direction of the tool (in the case of machining), the processing of these surfaces is demanding. Further research is needed to find optimal processing parameters for this type of material. Also, in order to better analyze and examine the surface of machined printed parts, it is worth comparing the quality of this surface with the machined surface of the cast material.

In recent years, more and more parts are manufactured using the SLM method in various industries (mainly in the aviation, automotive, and medical industries). SLM technology is a relatively new technology. Therefore, the analysis of the machinability of metallic printed material is new/innovative. The surface of printed parts requires improvements in quality—surface roughness. Hence, there is a need for effective manufacturing techniques that improve the quality of the side surfaces of printed parts. In our work we try to fill this research gap.

This work mainly focused on the comparative analysis of the quality of the machined surface of materials made of AISI 316L steel, produced using various methods. The processing of cast material and material produced using the additive method—selective laser melting (SLM)—was analyzed. The work analyzed the quality of the machined surface (surface roughness Ra) after the milling and electrical discharge machining (WEDM) of both types of material. In the case of the sintered material, the influence of the layer arrangement in the direction of the tool’s passage (perpendicular, parallel) was also analyzed. For milling tests, the Taguchi plan was adopted with three variable values of two parameters such as cutting speed and feed per revolution. For the WEDM process, the analyzed variable parameter was the current amplitude. The purpose of the experimental tests performed was to analyze the influence of variable process parameters on the quality of the machined surface. The work focused mainly on the analysis of the influence of machining parameters on the surface roughness—the roughness parameter Ra. In this work, the novelty is the comparison of the processed surface of materials manufactured using different methods (SLM, cast).

## 2. Materials and Methods

### 2.1. Material

For experimental tests of milling and wire electrical discharge machining, AISI 316L stainless steel was selected as the research material, which is determined by X2CrNiMo17-12-2/1.4404 according to European standards and includes the austenitic structure of stainless steel. The choice of 316L stainless steel as the workpiece material results from its wide use in various industries. The tests were performed on cast material and the material was laser-sintered using the selective laser melting (SLM) method. The sintered material was printed using a RENISHAW AM 250 Laser Melting System (Swords Business Park, Mountgorry, Swords, Co., Dublin, Ireland). DEW AS4-P/LC powder (DEUTSCHE EDELSTAHLWERKE GMBH, Krefeld, Germany) was used. Laser sintering took place in a protected atmosphere with argon. The choice of the SLM method to produce the processed material results from the presence of this technique in manufacturing for several years. The accuracy of the geometry of printed parts and their surface quality are still being tested.

Table 1 shows the parameters of 316L steel powder sintering using the SLM method. The chemical composition of this steel is presented in the Table 2. Table 3 presents selected thermo-physical and mechanical properties of this steel, which influence its processing.

### 2.2. Experimental Tests

**Figure 1:**
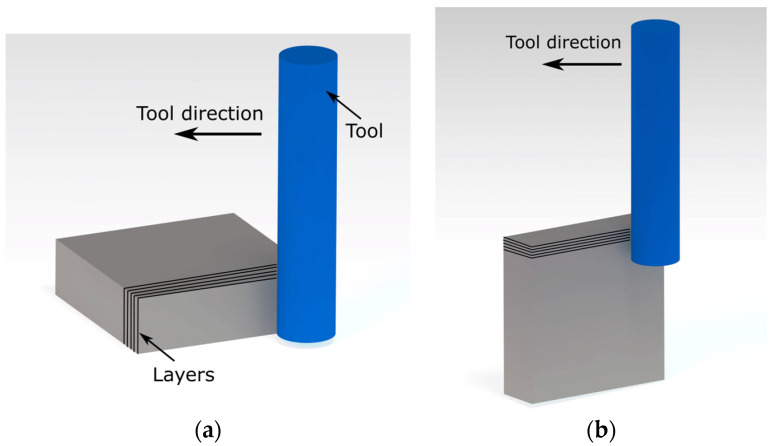
The direction of the tool transition to the direction of the sintered material layers: (**a**) perpendicular; (**b**) parallel.

In the experimental tests, cylindrical climb milling without cooling and the wire electro-discharge machining of cast and laser-sintered material using the SLM method were performed. The choice of the first method results from the widespread use of conventional machining. On the other hand, the WEDM technique ensures good dimensional and shape accuracy and enables the processing of modern engineering materials. In the case of machining sintered material, the machining was performed perpendicular and parallel to the arrangement of the sintered layers (Figure 1). In the case of sintered material, this approach was analyzed due to various surfaces requiring the additional processing of the parts after printing.

The processed surface (for milling and WEDM) was 8 mm long and 6 mm wide. The analyzed result factors were the quality of the machined surface. For this purpose, the influence of variable process parameters on the values of the roughness parameter Ra was analyzed. The surface roughness Ra value was measured with a Taysurf Intra 50 contour measuring tool (Taylor Hobson, Leicester, UK), equipped with a measurement tip with a rounding radius of 2 µm. Three surface roughness measurements were made on each surface over a 1 mm distance. Additionally, the treated surface was observed under a microscope—the VHX-600 digital microscope (Keyence, Osaka, Japan) at 500× magnification. The length and width of the chips were also measured using a microscope. Each measurement was performed three times and the calculated average values were selected for analysis.

**Figure 2:**
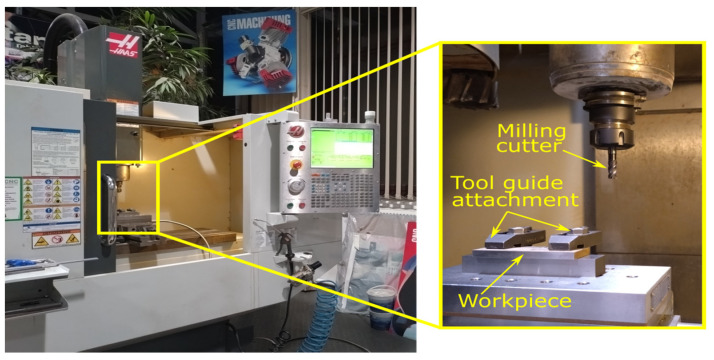
The experimental stand of milling process.

**Figure 3:**
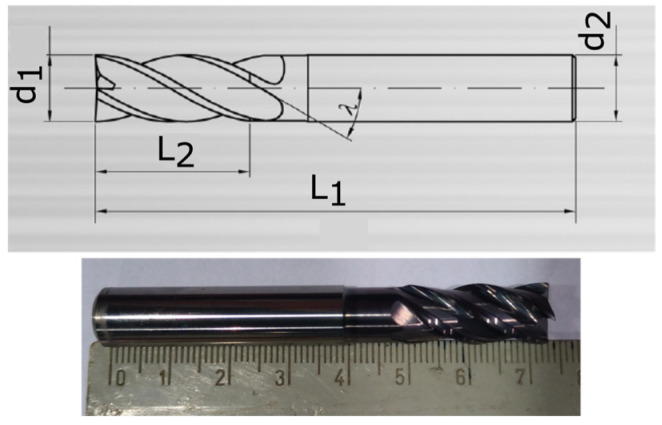
The scheme and photograph of milling cutter.

The milling process was performed on the Haas Minimill CNC machining (HAAS AUTOMATION, INC, Oxnard, CA, USA) center (Figure 2). For milling, a VHM carbide end mill with TiAIN coating was used with a working diameter of 10 mm, shank diameter of 10 mm, length of the working part of 25 mm, total length of 75 mm, angle *λ* = 33°, and 4 blades (Figure 3). A cutting depth of 0.4 mm was used. Additionally, during milling, the cutting force was measured for each test. The cutting force values were measured with the use of a Kistler 9257B piezoelectric dynamometer connected to a PC through a Kistler 5070B charge amplifier (Kistler Group, Winterthur, Switzerland). The PC was equipped with DynoWare software (Version 2825A, Kistler Group, Winterthur, Switzerland).

The milling test plan was performed according to the Taguchi plan. The Taguchi plan is often used to analyze machining results [24]. The variable parameters were cutting speed (*v_c_*) and feed per revolution (*f*). To determine the value of the feed, parameters with load per blade were adopted. Feed values were calculated based on the assumed spindle speed values: 955; 1910; 2865 rev/min, respectively, and the following feed speeds: 231; 462; 693 mm/min. The feed per revolution *f* values were calculated according to Formula (1):

feed (mm/rev) = feed speed/spindle speed.(1)

Table 4 shows the applied values of variable parameters along with the levels in the plan. Table 5 shows the Taguchi research plan used.

The statistical analysis was performed using the Minitab statistical software 22 (Minitab LLC., State College, PA, USA). In order to analyze the results and the influence of milling process parameters (*v_c_*, *f*) on the surface roughness Ra value, the Analysis of Variance (ANOVA) method was used. A linear regression function with coefficients in cutting speed *vc__xi_* and feed per revolution *f__xi_* was adopted. The function adopted the levels presented in Table 6.

Taking into account the coefficient α = 0.05, the adopted regression functions for the relationship between milling parameters and surface roughness Ra values have the following form: Table 7 presents the results of the ANOVA statistical analysis (DF—the total degrees of freedom, Adj SS—adjusted sums of squares, Adj MS—adjusted mean squares). A general linear model was chosen to generate the regression function. Below are the regression functions for the Ra surface roughness results for each milling type:

- cast material (cast):

*Ra* (*v_c_*, *f*) = 0.287 + 0.107 ∗ *v_c_*__30_ − 0.118 ∗ *v_c_*__60_ + 0.011 ∗ *v_c_*__90_ − 0.203 ∗ *f*__0.08_ − 0.006 ∗ *f*__0.12_ + 0.004 ∗ *f*__0.16_ + 0.062 ∗ *f*__0.24_ + 0.207 ∗ *f*__0.36_ − 0.022 ∗ *f*__0.48_ − 0.043 ∗ *f*__0.73_, (2)

- SLM-sintered material—milling in a direction perpendicular to the arrangement of layers (SLM perpendicular):

*Ra* (*v_c_*, *f*) = 0.277 − 0.047 ∗ *v_c_*__30_ − 0.075 ∗ *v_c_*__60_ + 0.122 ∗ *v_c_*__90_ − 0.209 ∗ *f*__0.08_ − 0.035 ∗ *f*__0.12_ − 0.101 ∗ *f*__0.16_
 + 0.084 ∗ *f*__0.24_ + 0.078 ∗ *f*__0.36_ + 0.199 ∗ *f*__0.48_ − 0.016 ∗ *f*__0.73_, (3)

- SLM-sintered material—milling in a direction parallel to the arrangement of the layers (SLM parallel):

*Ra* (*v_c_*, *f*) = 0.261 + 0.055 ∗ *v_c_*__30_ + 0.039 ∗ *v_c_*__60_ − 0.094 ∗ *v_c_*__90_ + 0.077 ∗ *f___*_0.08_ − 0.193 ∗ *f*__0.12_ − 0.011 ∗ *f*__0.16_
 + 0.046 ∗ *f*__0.24_ − 0.083 ∗ *f*__0.36_ + 0.154 ∗ *f*__0.48_ + 0.01 ∗ *f*__0.73_. (4)

**Figure 4:**
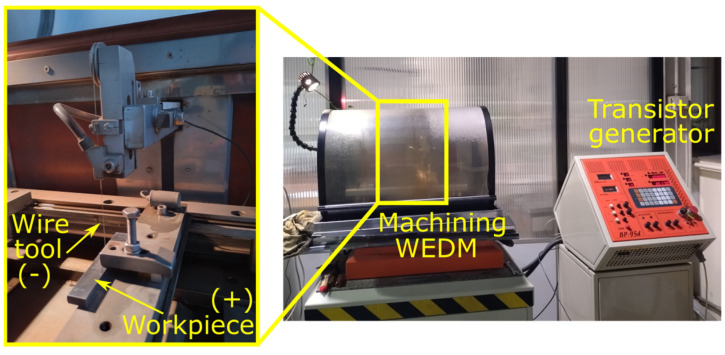
The WEDM test stand and the experimental setup.

Wire electrical discharge machining tests were performed on a BP95d electroerosion cutting machine (Zakład Automatyki Przemysłowej B.P., Końskie, Poland). Figure 4 shows the research stand with the experimental setup. The influence of the current intensity amplitude on the surface roughness Ra value was analyzed. The amplitude of the current varied in the range of 16–56 A (changed every 8 A). Three repeated tests were performed for each current amplitude value. Table 8 presents the values of the adopted constant parameters and process conditions. For this process, the efficiency of electrical discharge cutting was additionally analyzed—the material removal rate (MRR) was calculated from Formula (5).

Material removal rate (mm/s) = the cutting length/the cutting time.(5)

## 3. Results and Discussion

**Figure 5:**
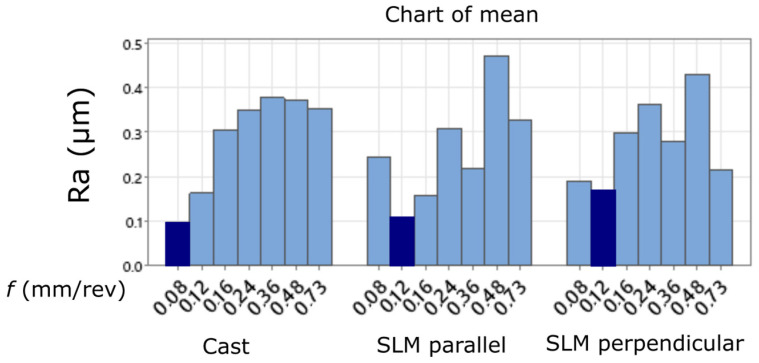
The influence of feed (*f*) on surface roughness (Ra) after milling.

Analyzing the obtained roughness measurement results, the lowest values of the Ra parameter were obtained for the milled surface. For the cast material, the lowest roughness of Ra = 0.10 µm was obtained for *v_c_* = 90 m/min and *f* = 0.08 mm/rev (the smallest value of the feed per revolution used). For the material manufactured using the SLM method, the lowest roughness Ra values were similar to those obtained for the cast material. For milling parallel to the layer arrangement, Ra = 0.11 µm, and for milling perpendicular to the layer arrangement, Ra = 0.17 µm. In both cases, the parameters *v_c_* = 60 m/min and *f* = 0.12 mm/rev were used (Figure 5). Moreover, the results for individual feed speeds show a different relationship between the roughness results for the sintered material compared to the cast material (Figure 5). For the cast material, the surface roughness decreases from a feed value of 0.24 mm/rev. However, for the sintered material, the roughness results in relation to the feed rate used are less predictable. It can be concluded that the method of producing the material affects the milling process. The structure of the material certainly has an influence here (layered and the presence of free spaces—pores), so the sintered material usually has a lower density compared to the cast material. Moreover, after the sintering process of metal powder, the thermo-physical properties of the material change. In the case of milling sintered material, the influence of the feed on the surface roughness results should be analyzed to a greater extent in order to select process parameters. The rest of the work presents a broader analysis of the influence of cutting speed and feed per revolution on surface roughness.

**Figure 6:**
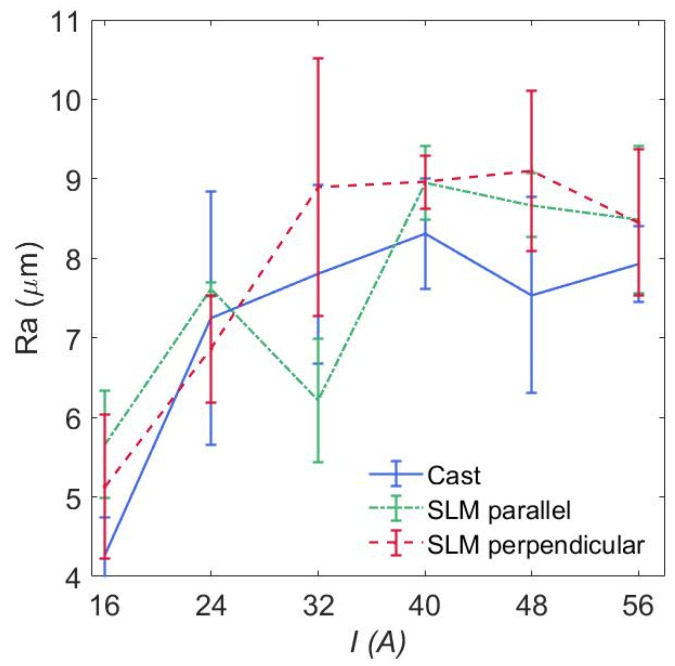
The influence of current amplitude (*I*) on surface roughness (Ra).

However, the surface after WEDM turned out to have a higher roughness than after the milling process. The purpose of the research was to check whether there are differences in the processing of AISI 316L steel produced using different methods. The obtained surface roughness after milling was in the range of 0.1–0.48 µm. However, after the WEDM process, the parameter Ra = 4.3–9.1 µm (Figure 6).

The analysis of surface roughness after WEDM did not show significant differences in the processing of the sintered material. For the EDM cutting of the sintered material and cast material, the surface roughness Ra value increases with the increase in the applied current amplitude, which is normal for this process (Figure 6).

In the case of the WEDM of both materials, the increase in surface roughness is, however, up to a certain value of the current amplitude used. A similar surface roughness was obtained for the *I* values used in the range of 32–56 A. This is due to the fact that WEDM is a less predictable process compared to machining. A further increase in roughness will not necessarily occur with an increase in the current amplitude. The analysis of the results shows that it is possible to reduce the energy consumption of the WEDM process while obtaining a satisfactory surface roughness. By using a current amplitude of 32–40 A, a similar surface roughness was obtained as when using *I* values of 48 A or 56 A.

**Figure 7:**
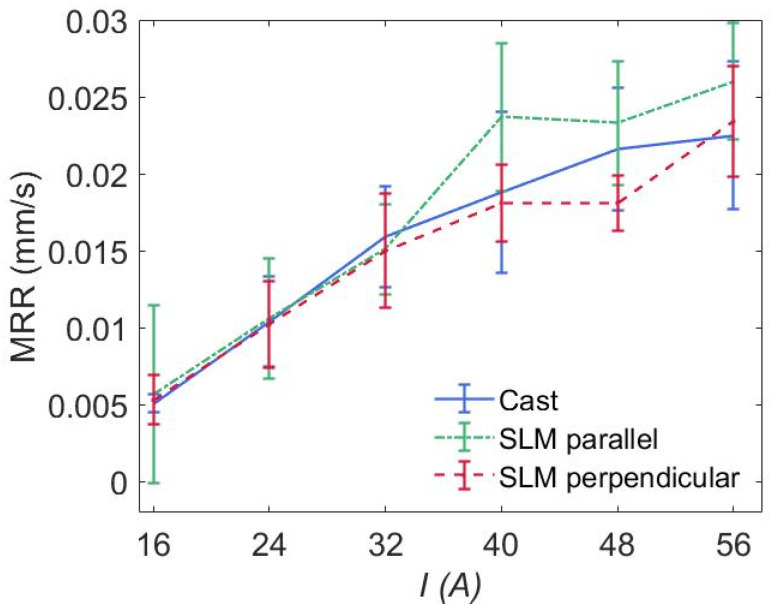
The influence of current amplitude (*I*) on material removal rate (MRR).

However, the surface roughness results in correlation with WEDM performance (Figure 7) show a rapid increase in the material removal rate for the application of *I* = 40 A. Further increasing the current amplitude results in a small increase in MRR. Hence, after taking into account the Ra roughness results, the value of 40 A can be considered optimal in this case.

**Figure 8:**
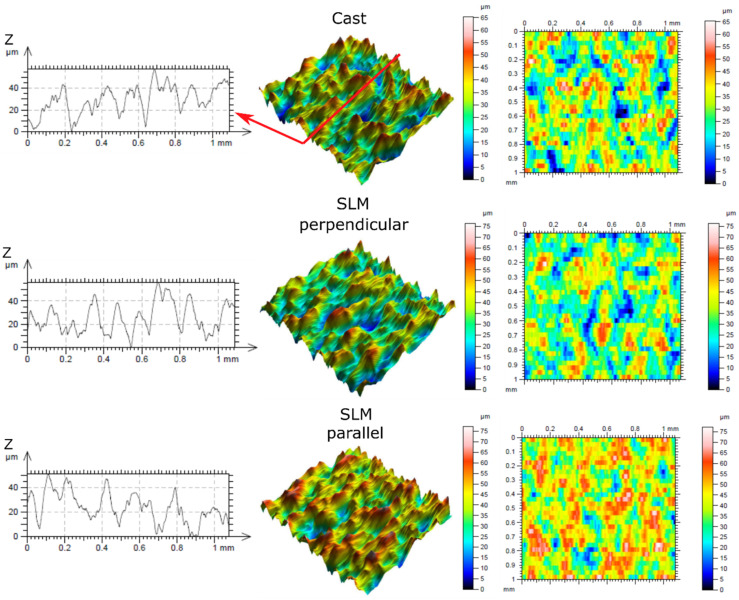
Topography of the surface after WEDM with using *I* = 40A.

To illustrate the surface after EDM cutting using *I* = 40 A, the analyzed types of machined surfaces are listed below (Figure 8). When machining sintered material in parallel layers, the surface topography appears rougher, but the profile analysis shows similar results for all machined surfaces.

**Figure 9:**
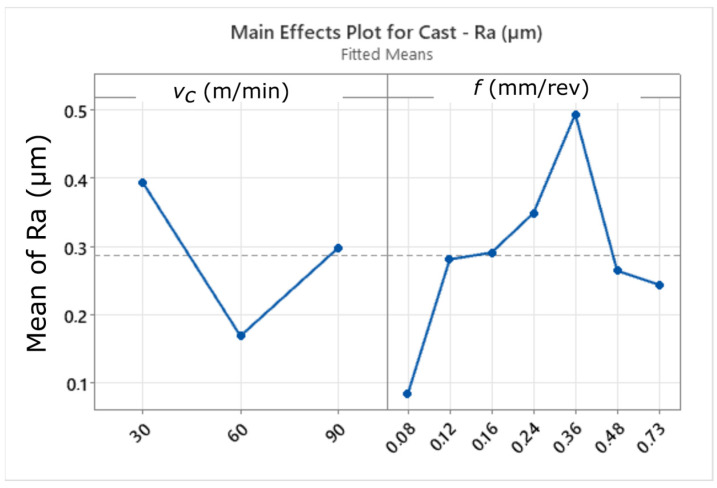
The influence of individual variables on the mean value of surface (Ra)—cast AISI 316L.

**Figure 10:**
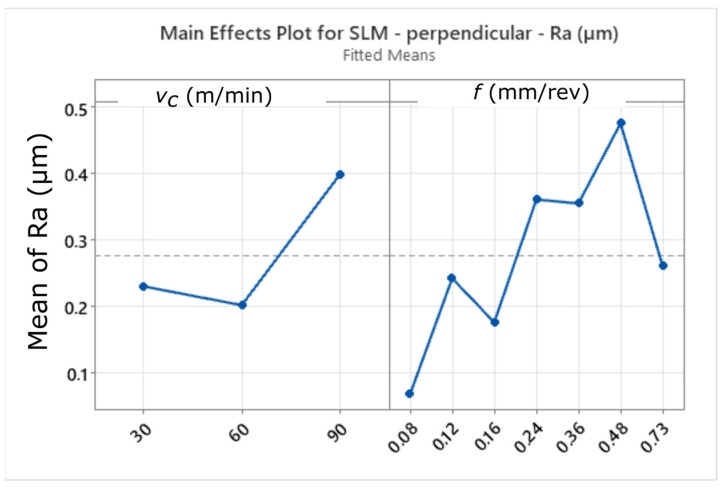
The influence of individual variables on the mean value of surface (Ra)—SLM perpendicular AISI 316L.

Much more information about the dependence of the surface roughness on machining parameters was provided by the analysis of the surface after the milling process. To check the influence of cutting speed (*v_c_*) and feed per revolution (*f*) on the roughness parameter Ra, the ANOVA method was used. The feed had the greatest impact on the Ra parameter. Here, the well-known relationship between a better surface quality and a reduced feed per revolution is visible. However, such a relationship occurs for the milling of cast material (Figure 9) and milling perpendicular to the arrangement of layers—printed material (Figure 10).

**Figure 11:**
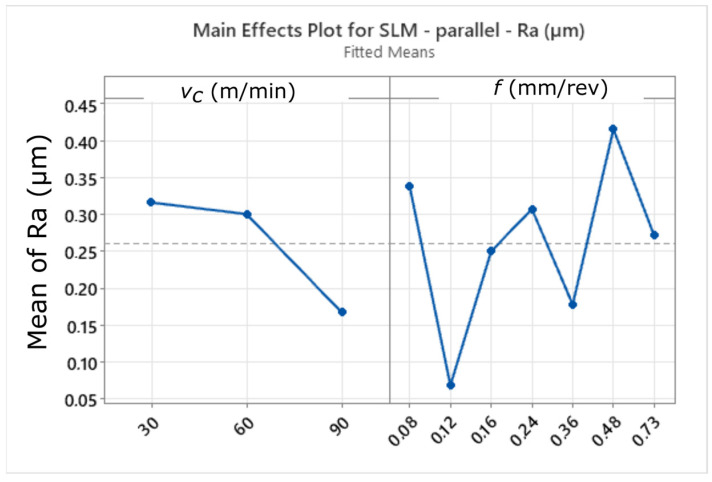
The influence of individual variables on the mean value of surface (Ra)—SLM parallel AISI 316L.

For milling cast and sintered material in a direction perpendicular to the layer arrangement, the lowest feed ensured the lowest surface roughness. However, for printed material milled parallel to the arrangement of layers, the effect of feed turned out to be ambiguous (Figure 11). These results show that the orientation of the sintered material layers significantly affects the results of the milling process. In [10], differences were also obtained in milling perpendicular and parallel to the direction of the layer arrangement.

**Figure 12:**
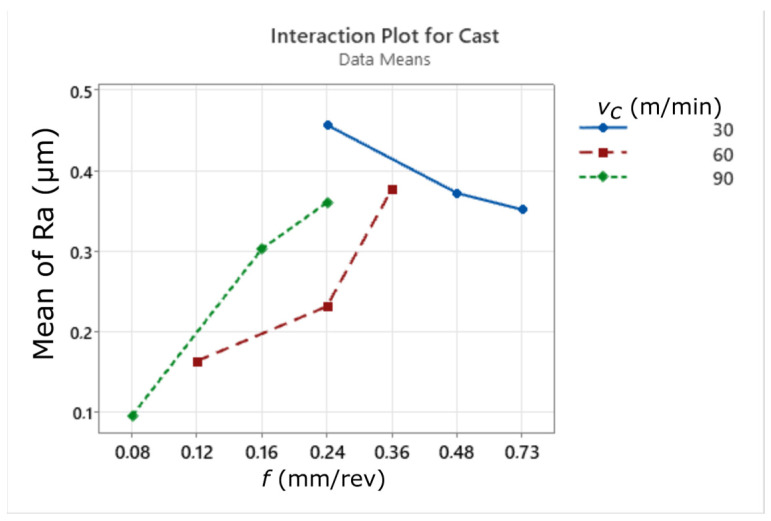
The influence of cutting speed and feed on the mean of Ra—cast AISI 316L.

**Figure 13:**
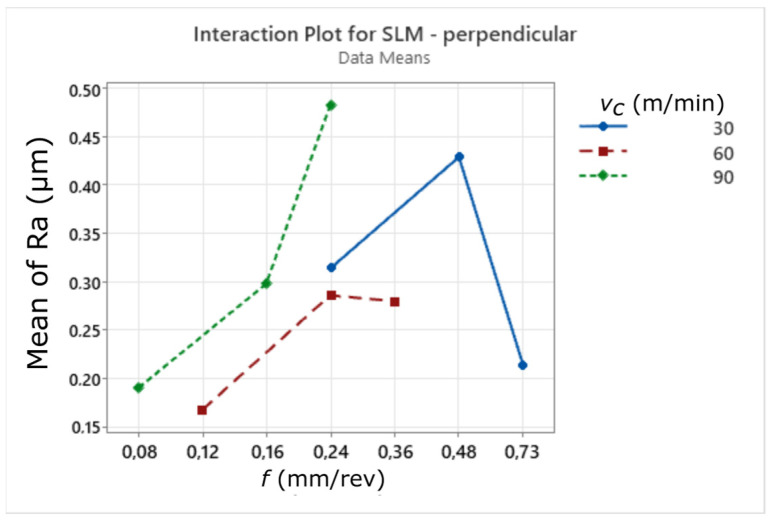
The influence of cutting speed and feed on the mean of Ra—SLM perpendicular AISI 316L.

The analysis of the influence of the cutting speed on the roughness parameter also showed similar results for the milled cast material and the sintered material in the direction parallel to the layer arrangement (Figure 12 and Figure 13). For this milling, cutting speeds of 60 m/min and 90 m/min gave similar Ra values and similar characteristics (better surface quality for lower f values). Certainly, machining with a higher cutting speed gives greater efficiency.

**Figure 14:**
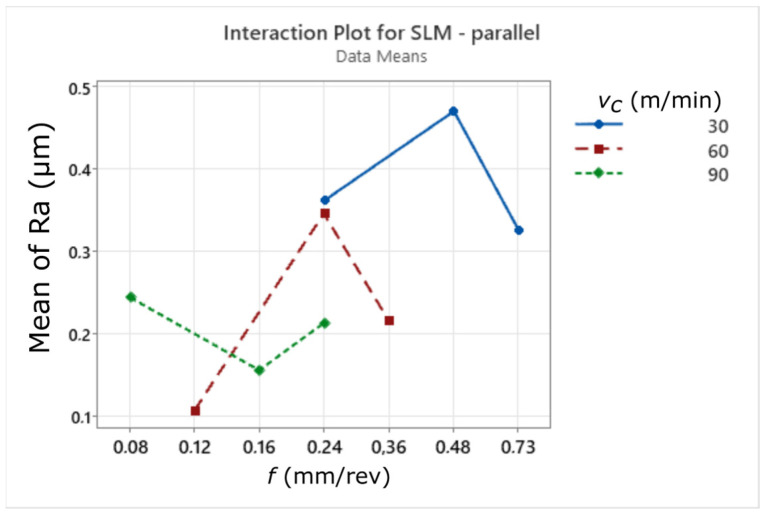
The influence of cutting speed and feed on the mean of Ra—SLM parallel AISI 316L.

**Figure 15:**
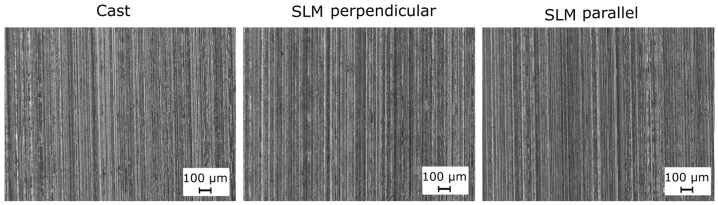
Photograph of machined surface using the following parameters: *v_c_* = 90 m/min and *f* = 0.08 mm/rev.

For milled cast material and sintered material milled perpendicularly to the arrangement of the layers, the most optimal values of *v_c_* = 60 m/min and *v_c_* = 90 m/min can be assumed with *f* = 0.12 mm/rev and *f* = 0.08 mm/rev, respectively. Then, the obtained surface roughness values are in the range of Ra = 0.1–0.2 µm. However, for milled sintered material that is processed parallel to the arrangement of layers (Figure 14), an ambiguous effect of cutting speed on the surface roughness is observed, as shown in Figure 11. In this case, the influence of cutting speed and feed on rotation is ambiguous. The photos below (Figure 15) show the milled surfaces with the most satisfactory surface roughness values. The surface is uniform with clear traces of the cutting blades.

**Figure 16:**
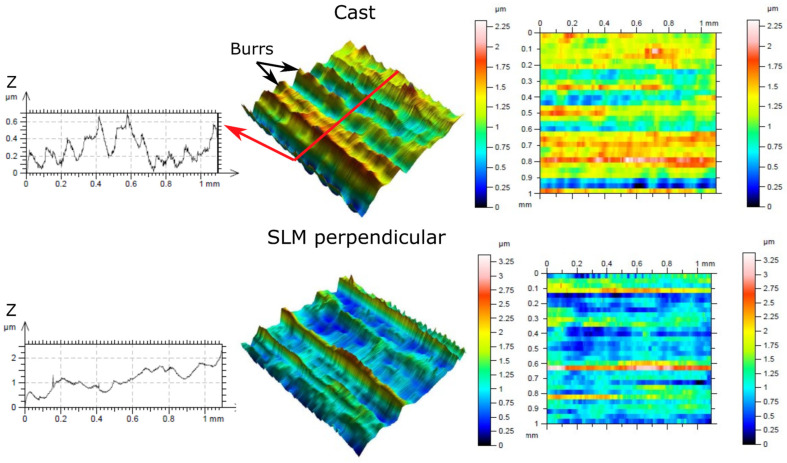

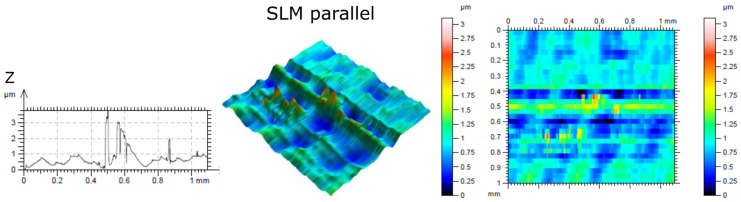
Topography of the three-dimensional surface after milling using the following parameters: *v_c_* = 90 m/min and *f* = 0.08 mm/rev.

Additionally, the analysis of the surface topography revealed the presence of burrs (Figure 16). The shape of the burrs on the machined surface of the cast material and sintered material milled perpendicular to the layer arrangement was analyzed. For these surfaces, defects in the form of burrs are also similar, unlike the burrs on the surface after milling parallel to the layer arrangement. In [10], it was shown that the dimensions of the material’s melting path influence the burrs that form. However, the influence of the feed direction on the layer arrangement was attributed to chip separation.

**Figure 17:**
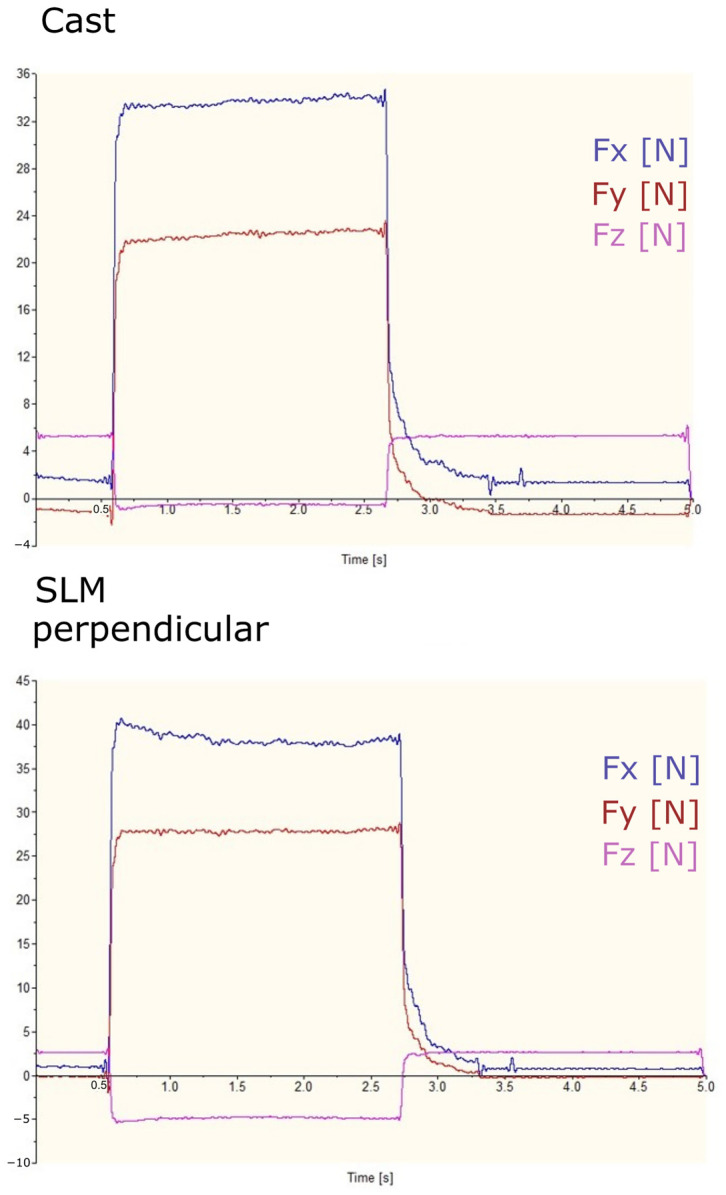

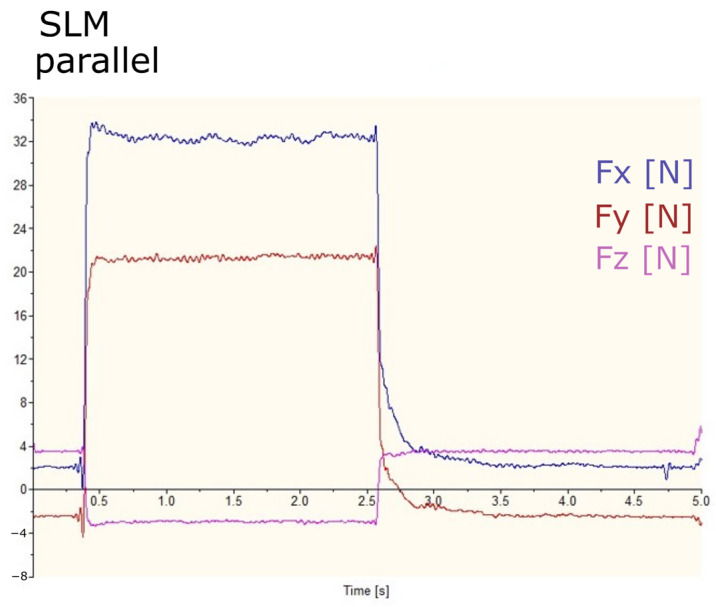
Measurement of the cutting force during milling using the following parameters: *v_c_* = 90 m/min and *f* = 0.08 mm/rev.

In the cutting force measurements shown below (Fx—resistive force, Fy—feed force, Fz—vertical force), similar force values are observed for the types of machining materials used for the parameters that give the best surface roughness results (Figure 17). The material production technique did not influence the forces occurring in the machining area during milling.

**Figure 18:**
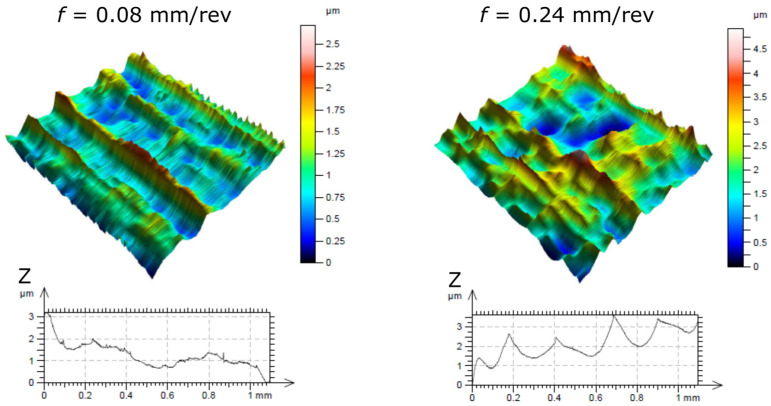
Topography of the surface three-dimensional after milling using the following parameters: *v_c_* = 90 m/min and various feed (*f*).

The ANOVA test showed the greatest influence on the surface roughness of the feed per revolution. Also, a comparison of the surface topography and the profile of the surface machined with feed rates *f* = 0.08 mm/rev and *f* = 0.24 mm/rev show higher burrs. Figure 18 shows this difference for a surface that was milled perpendicular to the arrangement of the sintered material layers. It can therefore be concluded that the geometric dimensions of the burrs depend mainly on the feed value. A similar conclusion was found in [14].

**Figure 19:**
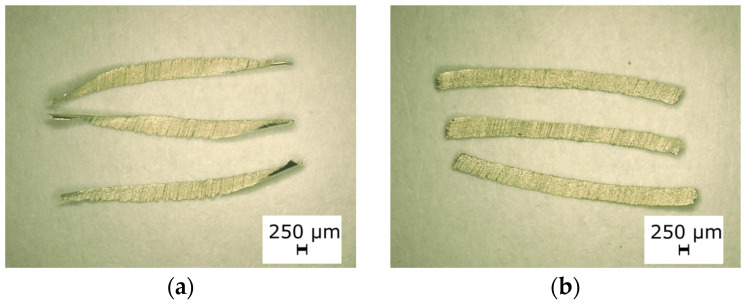
Photograph of cutter chips after milling in the perpendicular direction of the sintered material layers, using the following parameters: (**a**) *v_c_* = 90 m/min and *f* = 0.08 mm/rev; (**b**) *v_c_* = 60 m/min and *f* = 0.24 mm/rev.

The geometry (length, width) of the chips is similar for all samples. The chip width and length measurements showed that the selected parameters ensured chips of similar sizes. The measured width of the chips was in the range of 400–700 µm, while their length was approximately 7000 µm. The chips may be classified as acceptable. The values shown are average values from measuring a larger number of chips (10 chips). Moreover, most of the chips are flat, and there are chips with a slightly twisted geometry (Figure 19). The chips after milling with the parameters *v_c_* = 90 m/min, *f* = 0.08 mm/rev for all types of materials have a mainly slightly twisted shape.

**Figure 20:**
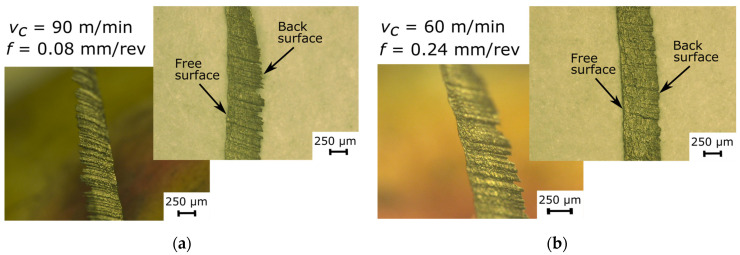
Photograph of cutter chips after milling in the perpendicular direction of the sintered material layers: (**a**) *v_c_* = 90 m/min and *f* = 0.08 mm/rev; (**b**) *v_c_* = 60 m/min and *f* = 0.24 mm/rev.

Additionally, we checked how the material was formed during milling on chips and their free surface and back surface. For a higher cutting speed (*v_c_* = 90 m/min, *f* = 0.08 mm/rev), the back surface of the chip turned out to be more jagged compared to the use of a lower cutting speed (*v_c_* = 60 m/min, *f* = 0.24 mm/rev) (Figure 20). This is due to the impact of the conditions in the processing area on the stainless steel. During machining, this steel hardens, making the chips more brittle for higher cutting speeds. This explains why the back face of the chip was more chipped when the cutting speed of 90 m/min was used. Additionally, the more chipped back face results from the slightly twisted chip geometry. When the chip was bent, chips formed more easily.

The above analysis showed that the direction of passage of the tool for arranging sintered layers during milling affects the surface roughness results. The analysis of the results showed similar surface roughness Ra values for the milling of the cast material and milling perpendicular to the arrangement of the layers of the sintered material. In this case, the best surface was obtained for milling with a low feed and higher cutting speed (values of these parameters: *f* = 0.08 mm/rev and *f* = 0.12 mm/rev and *v_c_* = 90 m/min and *v_c_* = 60 m/min, respectively). The feed turned out to have the greatest impact on the surface roughness. However, for WEDM, the machining of sintered material for the analyzed types showed similar results in surface roughness and the material removal rate. This is an important conclusion due to the possibility of using this method to improve the quality of various surfaces of SLM-printed material.

Moreover, both processing methods (milling, WEDM) can be used to improve the surface quality of parts printed using the SLM method. The milling process requires the adjustment of the processing parameters depending on the arrangement of the laser-sintered layers in the direction of the tool. Also, depending on the complexity of the part’s shape, the milling process may extend the finishing time. In these tests, the obtained surface roughness value for the WEDM process turned out to be higher compared to that obtained after milling. However, WEDM makes it possible to process parts with complex geometry. Most often, parts produced using SLM printing have a complex geometry. Of course, in further research, it is planned to expand the number of WEDM process parameters used to obtain lower surface roughness Ra values.

## 4. Conclusions

The study carried out a comparative analysis of the quality of processed surfaces of materials produced using various methods (selective laser melting, cast). The surface roughness Ra value after milling and wire electrical discharge machining was analyzed. In the case of the sintered material, the influence of the direction of the arrangement of sintered layers (perpendicular, parallel) to the direction of processing was also analyzed. The comparative analysis showed differences in the processing of selected materials and different effects of the direction of the layer arrangement on the processing. The following conclusions were drawn from the detailed analysis of the results:

- The comparative analysis showed a similar nature of the milling processing of the cast material and SLM-sintered material in the direction perpendicular to the arrangement of layers;
- The direction of arrangement of the SLM-sintered material layers to the direction of tool passage influences the surface roughness in milling. Laying the layers parallel to the direction of tool passage gives ambiguous roughness Ra value results;
- Milled surfaces gave similar roughness results for the machining of cast and sintered material processed perpendicular to the layer arrangement. For the feed of 0.12 mm/rev and a cutting speed of 60 m/min and a feed of 0.08 mm/rev, a roughness Ra value in the range of 0.1–0.2 µm was obtained;
- The feed per revolution parameter has the greatest impact on the surface roughness. The smaller the feed value, the lower the Ra value, but only when the *v_c_* = 60 m/min and *v_c_* = 90 m/min. When the *v_c_* = 30 m/min, the roughness results were ambiguous;
- The method of producing the material slightly affects electrical discharge cutting. The optimal value of the current amplitude was 40 A, obtaining a roughness Ra value of 8 µm and MRR of approximately 0.02 mm/s.

Further research should extend the analysis of the influence of the parallel arrangement of sintered layers to the tool direction on the roughness parameter. In subsequent research, there is a need to analyze the impact of the cutting tool wear, as well as to perform data simulations in order to optimize the data. However, for the WEDM analysis, an analysis of the influence of additional process parameters (pulse on time, operating voltage) should be performed. This is important due to the improvement of differently located SLM-printed surfaces.

## Figures and Tables

**Table 1:** The SLM process parameters.

Parameter, (Unit)	Value
Laser power, (W)	200
Layer thickness, (µm)	50
Powder distribution speed, (mm/s)	5000
Laser operation time, (µs)	80
Distance between sintering points, (µm)	60
Grain size, (µm)	20–53

**Table 2:** Chemical composition of AISI 316L powder (%) [21].

C	Si	Mn	Cr	Ni	Mo	Fe
<0.03	<1.0	<2.0	16.0–18.0	11.0–14.0	2.0–3.0	Ballance

**Table 3:** The selected thermo-physical [22] and mechanical properties of AISI 316L [23].

Property, (Unit)	Value
Density, (kg/m^3^)	7950
Specific heat capacity, (J/(kgK))	480
Melting point, (K)	1683
Thermal conductivity, (W/(mK))	15
Yield strength, (MN/m^2^)	280
Young’s modulus, (GN/m^2^)	211
Tensile, (MN/m^2^)	650

**Table 4:** The variables values in the experimental test.

No.	Coded Parameter	Actual Parameter	Value								
			1			2			3		
1.	A	*v_c_* (m/min)	30			60			90		
			1	2	3	1	2	3	1	2	3
2.	B	*f* (mm/rev)	0.24	0.48	0.73	0.12	0.24	0.36	0.08	0.16	0.24

**Table 5:** The Taguchi research plan.

Test No.	A	B	*v_c_* (mm/min)	*f* (rev/min)
1.	1	1	30	0.24
2.	1	2	30	0.48
3.	1	3	30	0.73
4.	2	1	60	0.12
5.	2	2	60	0.24
6.	2	3	60	0.36
7.	3	1	90	0.08
8.	3	2	90	0.16
9.	3	3	90	0.24

**Table 6:** Adopted levels of coefficient values.

Coefficient	Type	Levels Value of Coefficient—*xi*								
*v_c_xi_*	Fixed	1			2			3		
		30			60			90		
*f__xi_*	Fixed	1	2	3		4	5		6	7
		0.08	0.12	0.16		0.24	0.36		0.48	0.73

**Table 7:** The statistical results of ANOVA for Ra.

	Cast			SLM Perpendicular			SLM Parallel		
Source	DF	Adj SS	Adj MS	DF	Adj SS	Adj MS	DF	Adj SS	Adj MS
*v_c_* (m/min)	2	0.02542	0.01271	2	0.02269	0.01134	2	0.01335	0.006673
*f* (mm/rev)	6	0.06847	0.01141	6	0.07593	0.01265	6	0.04383	0.007305
Error	0	-		0			0		
Total	8	0.10657		8	0.08781		8	0.10348	

**Table 8:** The WEDM parameters and machining conditions [21].

Machining Parameter, (Unit)	Value/Characteristic
Pulse on time, (µs)	10
Pulse off time (µs)	350
The interelectrode gap size, (mm)	0.28
Wire feed rate, (mm/s)	10
Current amplitude, I (A)	16; 24; 40; 48; 56
Material of wire tool electrode	Brass
Wire tool diameter, (mm)	0.25
Working fluid, (-)	Demineralized water
Temperature of working fluid, (°K)	~294

## Data Availability

The original contributions presented in the study are included in the article, further inquiries can be directed to the corresponding author.
